# Effects of a Patient Portal Intervention to Address Diabetes Care Gaps: Protocol for a Pragmatic Randomized Controlled Trial

**DOI:** 10.2196/56123

**Published:** 2024-06-28

**Authors:** Amber J Hackstadt, Tom A Elasy, Sapna Gangaputra, Kryseana J Harper, Lindsay S Mayberry, Lyndsay A Nelson, Neeraja B Peterson, S Trent Rosenbloom, Zhihong Yu, William Martinez

**Affiliations:** 1 Department of Biostatistics Vanderbilt University Medical Center Nashville, TN United States; 2 Division of General Internal Medicine Department of Medicine Vanderbilt University Medical Center Nashville, TN United States; 3 Department of Ophthalmology and Visual Sciences Vanderbilt University Medical Center Nashville, TN United States; 4 Department of Biomedical Informatics Vanderbilt University Medical Center Nashville, TN United States

**Keywords:** patient portals, self-management, self-efficacy, diabetes mellitus, health literacy, attitudes

## Abstract

**Background:**

Despite the potential to significantly reduce complications, many patients do not consistently receive diabetes preventive care. Our research team recently applied user-centered design sprint methodology to develop a patient portal intervention empowering patients to address selected diabetes care gaps (eg, no diabetes eye examination in last 12 months).

**Objective:**

This study aims to evaluate the effect of our novel diabetes care gap intervention on completion of selected evidence-based diabetes preventive care services and secondary outcomes.

**Methods:**

We are conducting a pragmatic randomized controlled trial of the effect of the intervention on diabetes care gaps. Adult patients with diabetes mellitus (DM) are recruited from primary care clinics affiliated with Vanderbilt University Medical Center. Participants are eligible if they have type 1 or 2 DM, can read in English, are aged 18-75 years, have a current patient portal account, and have reliable access to a mobile device with internet access. We exclude patients with medical conditions that prevent them from using a mobile device, severe difficulty seeing, pregnant women or women who plan to become pregnant during the study period, and patients on dialysis. Participants will be randomly assigned to the intervention or usual care. The primary outcome measure will be the number of diabetes care gaps among 4 DM preventive care services (diabetes eye examination, pneumococcal vaccination, hemoglobin A_1c_, and urine microalbumin) at 12 months after randomization. Secondary outcomes will include diabetes self-efficacy, confidence managing diabetes in general, understanding of diabetes preventive care, diabetes distress, patient portal satisfaction, and patient-initiated orders at baseline, 3 months, 6 months, and 12 months after randomization. An ordinal logistic regression model will be used to quantify the effect of the intervention on the number of diabetes care gaps at the 12-month follow-up. For dichotomous secondary outcomes, a logistic regression model will be used with random effects for the clinic and provider variables as needed. For continuous secondary outcomes, a regression model will be used.

**Results:**

This study is ongoing. Recruitment was closed in February 2022; a total of 433 patients were randomized. Of those randomized, most (n=288, 66.5%) were non-Hispanic White, 33.5% (n=145) were racial or ethnic minorities, 33.9% (n=147) were aged 65 years or older, and 30.7% (n=133) indicated limited health literacy.

**Conclusions:**

The study directly tests the hypothesis that a patient portal intervention—alerting patients about selected diabetes care gaps, fostering understanding of their significance, and allowing patients to initiate care—will reduce diabetes care gaps compared with usual care. The insights gained from this study may have broad implications for developing future interventions to address various care gaps, such as gaps in cancer screening, and contribute to the development of effective, scalable, and sustainable approaches to engage patients in chronic disease management and prevention.

**Trial Registration:**

ClinicalTrials.gov NCT04894903; https://classic.clinicaltrials.gov/ct2/show/NCT04894903

**International Registered Report Identifier (IRRID):**

DERR1-10.2196/56123

## Introduction

### Background

Diabetes is a prevalent chronic condition requiring effective monitoring and preventive services to avoid costly and potentially severe complications such as cardiovascular and kidney diseases [1]. Unfortunately, many patients do not consistently receive evidence-based diabetes monitoring and preventive services [2,3]. For instance, despite the potential to significantly reduce severe vision loss, approximately 40% of Americans with diabetes fail to undergo a recommended annual eye examination [2,3].

Previous studies have identified several barriers to accessing evidence-based diabetes monitoring and preventive services. These barriers encompass patient-related factors, such as limited health literacy and lack of awareness, as well as clinician- and system-related factors, such as time constraints and insufficient patient support between visits [4-8]. Previous attempts to improve the uptake of these services have yielded only modest results [9-11]. To achieve optimal rates of these services, there is an urgent need for an intervention that improves clinical efficiency, caters to patients with varying health literacy levels, and can be scaled and sustained effectively [4].

Patient portals present a promising platform to enhance access to services and support self-management by offering convenient health data tracking, educational resources, notifications, and improved patient-clinician communication, with the potential to address the limitations of costly and less scalable face-to-face interventions [12-16]. Recent advancements in patient portal technology have provided the infrastructure necessary for patients to self-schedule appointments and request health services, offering new opportunities to engage patients in their care [17-19]. Unfortunately, the varying design and usability of patient portals have resulted in inconsistent levels of patient engagement and impacts on diabetes care [12,20].

Recent research has demonstrated that patient-facing technology, enabling patients to self-order tests, can increase the uptake of colon cancer screening, enhance patient activation, and improve clinical efficiency by reducing the workload on clinicians [18,19]. In our previous work [21], our research team used the user-centered design sprint methodology to develop a patient portal intervention to address diabetes care gaps by notifying patients when any of the following four selected, evidence-based diabetes monitoring and preventive services [22] are due: (1) diabetes eye examination, (2) hemoglobin A_1c_, (3) urine microalbumin, and (4) pneumococcal vaccination; fostering understanding of the importance of these services through literacy-level appropriate content (eg, educational content that may be more easily understood by patients with limited health literacy); and allowing patients to initiate an order for the corresponding care.

In a pilot study of this diabetes care gap intervention (DCGI), patients highly rated the overall system usability, and more than 80% of patients indicated that they would continue to use the system in the future [23].

### Objective

This study aims to evaluate the effect of our novel DCGI on the completion of selected evidence-based, diabetes monitoring and preventive services and secondary outcomes (eg, diabetes self-efficacy).

## Methods

### Setting

For this study, we enrolled patients from 14 adult primary care clinics affiliated with Vanderbilt University Medical Center (VUMC) located throughout the Nashville, Tennessee, metropolitan area. The clinical data of these patients are stored in an electronic health record (EHR) system provided by Epic Systems Corp. To facilitate patient access to their clinical information, VUMC has implemented a widely adopted patient portal called My Health at Vanderbilt (MHAV) using Epic’s MyChart platform [24]. This patient portal is accessible both via a web-based app and dedicated mobile app compatible with iOS and Android operating systems.

### Study Design

This study is a 12-month pragmatic randomized controlled trial (RCT) with a 2-arm, parallel design. Participants assigned to arm 1 (usual care) have access to the standard version of MHAV, the patient portal. Participants in arm 2 have access to a modified version of MHAV that incorporates the DCGI described in detail below. Both groups of participants were informed that the study aims to assess satisfaction with 2 versions of MHAV among individuals with diabetes. Throughout the trial, the participants are requested to complete study questionnaires at 4 designated time points: T0 (baseline), T1 (3 months), T2 (6 months), and T3 (12 months).

The study protocol is guided by the principles outlined in the CONSORT Statement: Extension to Pragmatic Trials [25]. Consistent with pragmatic trial design, this study is designed to evaluate the real-world effectiveness of the DCGI in routine practice settings [26]. Due to the nature of the study and intervention, participants are aware of the intervention they are receiving, and the research team is not blinded to participant randomization.

### Eligibility and Recruitment

Participants were eligible if they received care at a participating VUMC clinic site and had type 1 or type 2 diabetes mellitus (patient reported and EHR verified), could read in English, were aged 18-75 years, had a current MHAV account, and had reliable access to a mobile device with the iOS or Android operating system with internet access. We excluded patients with a medical condition that prevents them from using a mobile device, severe difficulty seeing, pregnant women or women who plan to become pregnant during the study period, and patients on dialysis.

On a rolling basis, potentially eligible patients were selected from a randomly ordered list of established adult patients with diabetes from participating clinic sites and sent a recruitment letter describing the study. To facilitate a diverse study population representative of the population of patients with diabetes, we oversampled patients from minoritized groups. In addition, we sent secure messages via the patient portal to potentially eligible patients. To increase the diversity of study participants, we made follow-up phone calls to patients from minoritized groups who did not respond to the recruitment letter. Interested patients could contact a research assistant to learn more about the study. To enroll, participants reviewed a web-based study eligibility prescreener listing the study’s inclusion and exclusion criteria and completed a web-based electronic consent form via REDCap (Research Electronic Data Capture; version 5.0.8; Vanderbilt University).

### Procedures and Randomization

Study staff contacted all patients who completed a web-based electronic consent form to review study procedures, answer remaining questions, and confirm eligibility criteria. Enrolled participants were sent a baseline questionnaire via REDCap. After receiving the completed baseline questionnaire, the study coordinator (KJH) randomly assigned the participant to one of two groups: (1) intervention or (2) usual care, using REDCap’s randomization module. The randomization sequence was generated by the research team biostatistician (AJH) using a stratified, permuted, block randomization with block sizes of 4. To obtain balance across treatment groups on key variables, the randomization scheme was stratified by (1) 6 clinic groups (clinic sites with similar patient demographics were grouped together), (2) adequate versus limited health literacy, (3) whether or not the participant was from a minoritized racial or ethnic group, and (4) whether or not the participant currently had a diabetes care gap (ie, whether or not the patient had at least 1 relevant diabetes-related “health maintenance” topic within Epic’s EHR noted as overdue). Once the randomization assignment was finalized, participants in either arm received an email with their treatment assignment and an explanation of how to navigate to features of MHAV specific to their treatment group. A participant could withdraw from the study at any time by notifying the study team. In addition, participants were administratively withdrawn from the study by the investigators if they did not complete the baseline questionnaire needed for randomization.

### Intervention and Control

Participants randomized to the intervention arm were provided access to a version of MHAV embedded with the DCGI. This included patient portal notifications to patients with reminders for timely completion when any of the following four diabetes care gaps existed: (1) no diabetes eye examination in the last 12 months; (2) no prior pneumococcal vaccination (ie, never received PPSV-23, PCV-13, PCV-15, or PCV-20); (3) no hemoglobin A_1c_ in the last 6 months; and (4) no urine microalbumin in the last 12 months. Also included were associated literacy-level appropriate content to foster understanding of the importance of these services and accompanying functionality allowing patients to initiate an order for the corresponding care via the patient portal and then receive a confirmation message with instructions for obtaining the care and next steps.

**Figure 1:**
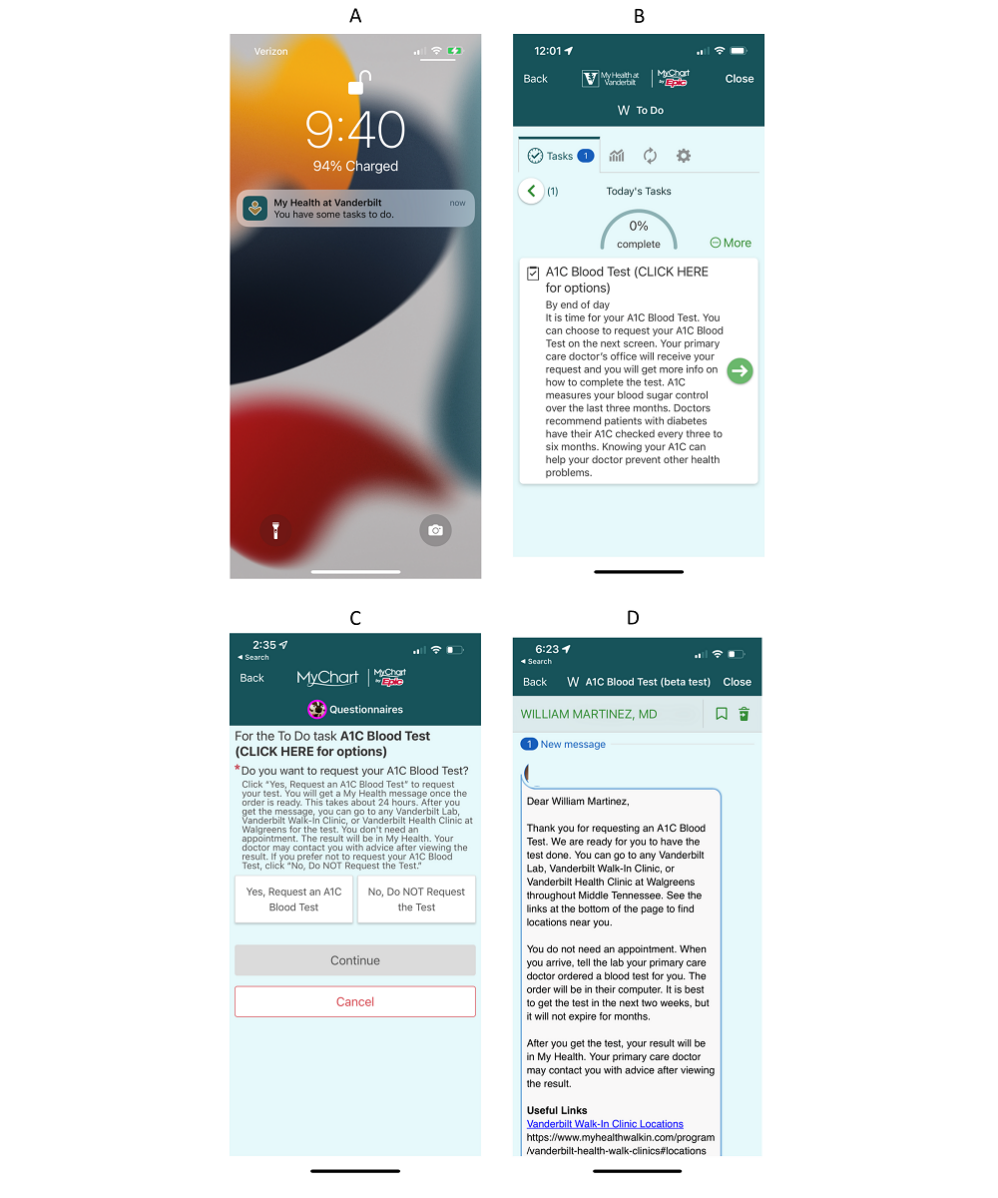
Example screenshots (from a smartphone) of the diabetes care gap intervention. (A) Initial push notification directing patient to their to-do list. (B) To-do item alerting patient about the care gap and its significance. (C) Patient-facing options for addressing the care gap. (D) Confirmation message to patients to initiate care, outlining next steps.

Since a considerable proportion of VUMC patients receive eye care outside of the Vanderbilt health system, the intervention also allows participants to indicate whether they received a diabetes eye examination in the last 12 months at a non-Vanderbilt clinic. In response to a diabetes care gap notification, patients can also indicate their preference to decline to self-initiate an order for the corresponding care (eg, patients with upcoming clinic appointments at which they expect to receive the care). A detailed description of the development of the intervention is available [21]. Figure 1 shows example screenshots of intervention components.

The DCGI uses Epic’s “health maintenance” topics to identify diabetes care gaps and trigger corresponding to-do list items within MHAV and associated push notifications (Figure 1A). Patients who have unresponded items on their To-Do list receive a reminder message via patient portal secure messaging approximately every 2 weeks. To allow patients to self-initiate orders for care, the to-do list items repurpose Epic’s questionnaire functionality to inform patients of diabetes care gaps and communicate their importance using language at or below a sixth grade reading level (Figure 1B). On the subsequent response screen, the to-do list item provides an “order” button that patients can click to initiate an order for the corresponding care (Figure 1C). Using Epic’s Reporting Workbench, care coordination nurses identify all patients requesting care via the intervention and generate bulk orders for the corresponding care. Patients are promptly notified with a confirmation message containing detailed instructions on the subsequent steps (Figure 1D). Associated orders are routed to the primary care physicians’ cosign basket for single-click cosignature within Epic’s EHR (Epic Systems Corporation), and associated results and eye examination findings are transmitted to the primary care physician via the EHR as usual. Regardless of the presence of any diabetes care gaps, patients in the intervention group receive a monthly study-related to-do list item to acknowledge in order to create familiarity with the feature and ensure that their to-do list is functioning properly.

Participants assigned to the usual care group have access to the existing version of MHAV, enabling them to access and review relevant health data, medical information related to their conditions, and engage in communication with their health care team. Randomized participants who do not show evidence of accessing the patient portal (control group) or accessing the study intervention within the patient portal (intervention group) are contacted within the first 6 months of study participation to ensure that they are not experiencing any technical issues and confirm treatment fidelity for both groups.

### Data Collection and Measures

#### Overview

Study participants complete questionnaires electronically via email using REDCap at 4 time points: baseline (T0), 3-month follow-up (T1), 6-month follow-up (T2), and 12-month follow-up (T3). The baseline questionnaire (T0) includes basic demographic questions and validated measures of health literacy [27], numeracy [28], and eHealth literacy [29]. Health literacy is assessed with a 1-item screener asking participants to rate their confidence independently completing medical forms. Consistent with prior research, participants noting any lack of confidence are classified as having limited health literacy [27,30,31]. Numeracy is assessed using the 8-item subjective numeracy scale with scale scores ranging from 1 (worst) to 6 (best) [28,32]. eHealth literacy is assessed by the 8-item eHealth Literacy Scale with scale scores ranging from 8 (worst) to 40 (best) [29]. We estimated the time to completion for each questionnaire to be about 20 minutes.

The primary outcome measure is the number of diabetes care gaps among 4 selected diabetes mellitus monitoring and preventive services at 12 months. Thus, the number of diabetes care gaps per patient can range from 0 to 4. Secondary intermediate outcomes include diabetes self-efficacy, attitudes toward managing diabetes in general, understanding of diabetes monitoring and preventive care, diabetes distress, satisfaction with MHAV, patient-initiated orders, and reported completion of diabetes eye examinations outside the VUMC system. Additional secondary outcomes, blood glucose control and treatment intensification, are assessed by EHR abstraction.

#### Primacy Outcome

##### Number of Diabetes Care Gaps at 12 Months

The number of diabetes care gaps per patient will be assessed via data abstraction from structured fields within the VUMC EHR. These data may include care received outside of the VUMC system and entered into the VUMC EHR by clinicians or patients. Consistent with diabetes evidence-based guidelines [22], the following diabetes care gaps will be assessed and defined as follows: (1) the diabetes eye examination gap, when there has been more than 12 months since the most recent diabetes eye examination date recorded in the EHR; (2) the pneumococcal vaccination gap, when there no record of any pneumococcal vaccination (ie, PPSV-23, PCV-13, PCV-15, or PCV-20) in the EHR; (3) the hemoglobin A_1c_ gap, it has been more than 6 months since the most recent hemoglobin A_1c_ result date in the EHR; and (4) the urine microalbumin gap, when it has been more than 12 months since the most recent urine microalbumin result date in the EHR.

Therefore, this outcome is ordinal, and participants can have between 0 (best) and 4 (worst) diabetes care gaps.

#### Secondary Outcomes

##### Number of Diabetes Care Gaps at 6 Months

The number of diabetes care gaps per patient at 6 months will be assessed and defined as above for the 12-month time point.

##### Diabetes Self-Efficacy

The Perceived Diabetes Self-Management Scale will be used to assess diabetes-specific self-efficacy [33]. The unidimensional, 8-item, validated scale is scored on a 5-point Likert scale. The total Perceived Diabetes Self-Management Scale score can range from 8 to 40, with higher scores indicating greater diabetes self-efficacy.

##### Confidence in Managing Diabetes in General

The Manage Disease in General Scale of the Chronic Disease Self-Efficacy Scales will be used to assess confidence toward managing diabetes in general [34]. The 5-item scale is a validated measure of the confidence a person has in managing his or her own health and health care. The items were adapted to be specific to diabetes rather than a generic chronic health condition or illness. Each item uses a 10-point Likert-type scale of response options ranging from 1 (not at all confident) to 10 (totally confident). The score for the scale is the mean of the items with scale scores ranging from 1 to 10. Higher scores indicate greater confidence toward managing diabetes in general.

##### Understanding of Diabetes Monitoring and Preventive Care

Unique study-specific items (4 items) that assess participants’ understanding of the recommended frequency of selected diabetes monitoring and preventive services (eg, diabetes eye examinations) will be administered to all study participants. Each multiple-choice item has only 1 correct answer and item responses are scored as “correct” or “incorrect.”

##### Diabetes Distress

The Problem Areas in Diabetes Scale (PAID-5) will be used to assess diabetes-related distress [35]. The 5-item, unidimensional scale is scored on a 5-point Likert scale ranging from 0=“not a problem” to 4=“serious problem.” Scale scores range from 0 to 20, with higher scores indicating greater diabetes-related emotional distress.

##### Satisfaction With MHAV

Satisfaction with MHAV will be assessed using the 10-item System Usability Scale, which queries users’ perceptions of ease of use, the likability of the interface, and overall satisfaction. The System Usability Scale uses a 5-point Likert scale from strongly disagree to strongly agree [36]. Participants’ responses to each item are scored 0-4, added together and then multiplied by 2.5 to generate a total score for the scale from 0 (worst) to 100 (best). In addition, unique study-specific items will assess acceptability (ie, likelihood of recommending to other patients and continuing to use) and open-ended items to describe elements participants liked best as well as challenges or problems they encountered and suggested improvements.

##### Patient-Initiated Orders

Among patients in the intervention group, the number of patient-initiated orders for each evidence-based diabetes monitoring and preventive service will be assessed by querying the EHR (eg, patient-initiated A_1c_ orders).

##### Reported Completion of Diabetes Eye Examinations Outside VUMC System

Among patients in the intervention group, the number of patient-reported diabetes eye examinations completed outside the VUMC system will be assessed by querying the EHR (ie, number of reports of diabetes eye examinations completed outside the VUMC system that are submitted in response to the To-Do item received via the DCGI). For each patient-reported diabetes eye examination outside the VUMC system, study staff will seek permission from participants to obtain the associated medical records. To assess the accuracy of patients’ self-report, the number of outside diabetes eye examination records that include screening for diabetic eye disease will be assessed by medical record review by trained ophthalmology staff and overseen by an ophthalmologist (SG).

##### Glycemic Control

The change in glycemic control will be assessed by abstracting, from the VUMC EHR, the closest hemoglobin A_1c_ measurement to the study time points: T0, T1, T2, and T3. For the baseline time point (T0), the date and value closest to the study time point in the window of 90 days prior to the study point will be selected. For all subsequent study time points (T1, T2, and T3), the date and value closest to the study time point in the window of 28 days prior to the study time point up until 28 days after the study time point will be selected.

##### Treatment Intensification

Treatment intensification for antihyperglycemic and antihypertensive medications will be assessed by abstracting medication history from the EHR. Treatment intensification will be defined as the addition or increase in dose of (1) antihyperglycemic and (2) antihypertensive medications, respectively.

### Data Analysis

#### Statistical Analysis Plan

The pragmatic, parallel design, RCT is designed to evaluate the effect of the intervention on diabetes care gaps as well as secondary intermediate outcomes (eg, diabetes self-efficacy). An ordinal logistic regression model will be used to quantify the effect of the patient portal intervention on the primary outcome of the number of diabetes care gaps at 12-month follow-up. For dichotomous secondary outcomes (eg, treatment intensification), a logistic regression model will be used with random effects for the clinic and provider variables as needed. For continuous secondary outcomes (eg, diabetes self-efficacy), a generalized linear regression model will be used. If there is imbalance, we will control for baseline patient-level covariates. We will allow for nonlinear associations by modeling continuous covariates with restricted cubic splines as needed. The characteristics of participants who do not complete the study will be compared for both study groups.

#### Primary Analysis

We will examine the impact of the DCGI on the number of selected diabetes care gaps at 12-month follow-up compared with the control condition (Table 1). We hypothesize that the patient portal intervention will facilitate the completion of selected evidence-based, diabetes monitoring and preventive services resulting in fewer diabetes care gaps. Analyses will be conducted using R statistical software (version 4.1.0 or higher; R Development Core Team).

**Table 1:** Outcomes and measures.

		Measures		Variable type		How collected		Time points^a^
**Primary outcome**								
	Number of diabetes care gaps at 12 months	Number of diabetes care gaps per patient out of 4 possible: no diabetes eye examination in the last 12 months; No prior pneumonia vaccinations (ie, never received PPSV-23, PCV-13, PCV-15, or PCV-20); No hemoglobin A_1C_ blood test in the last 6 months; No urine microalbumin in the last 12 months	Ordinal		EHR^b^ abstraction		T0-T3	
**Secondary outcomes**								
	Number of diabetes care gaps at 6 months	Number of diabetes care gaps per patient out of 4 possible: no diabetes eye examination in the last 12 months; no prior pneumonia vaccinations (ie, never received PPSV-23, PCV-13, PCV-15, or PCV-20); no hemoglobin A_1C_ blood test in the last 6 months; no urine microalbumin in the last 12 months	Ordinal		EHR abstraction		T0-T2	
	Diabetes self-efficacy	Perceived Diabetes Self-Management Scale [33]	Continuous		Questionnaire		T0-T3	
	Confidence toward managing diabetes in general	Items adapted from the Manage Disease in General Scale of the Chronic Disease Self-Efficacy Scales [34]	Continuous		Questionnaire		T0-T3	
	Understanding of diabetes monitoring and preventive care	Unique study-specific items to assess participants’ understanding of recommended diabetes monitoring and preventive care	Dichotomous		Questionnaire		T0-T3	
	Diabetes distress	Problem Areas in Diabetes Scale (PAID-5) [35]	Continuous		Questionnaire		T0-T3	
	Satisfaction with MHAV^c^	System Usability Scale [36] and user experience questions	Continuous		Questionnaire		T0-T3	
	Patient-initiated orders	Number of patient-initiated orders for evidence-based diabetes monitoring and preventive services (eg, hemoglobin A_1C_)	Continuous		EHR abstraction, tableau and clarity servers		T3	
	Reported completion of diabetes eye examination outside VUMC^d^ system	Number of reports of diabetes eye examinations completed outside the VUMC system in response to the to-do item received via the intervention	Continuous		EHR abstraction, tableau and clarity servers		T3	
	Change in glycemic control	Hemoglobin A_1C_	Continuous		EHR abstraction		T0-T3	
	Treatment intensification	Addition or increase in dose of: antihyperglycemic medications and antihypertensive medications	Dichotomous		EHR abstraction		T0-T3	

^a^T0: baseline, T1: 3-month follow-up, T2: 6-month follow-up, and T3: 12-month follow-up.

^b^EHR: electronic health record.

^c^MHAV: My Health at Vanderbilt.

^d^VUMC: Vanderbilt University Medical Center.

#### Secondary Analysis

In addition, we will quantify the effects of the DCGI on secondary intermediate outcomes including diabetes self-efficacy, confidence toward managing diabetes in general, understanding of diabetes monitoring and preventive services, diabetes distress, and satisfaction with MHAV (Table 1). Additional secondary outcomes including the number of patient-initiated orders, reported completion of diabetes eye examinations outside VUMC system, blood glucose control, and treatment intensification will also be assessed by EHR abstraction.

#### Sample Size and Power

The targeted enrollment for the study is 250 patients per treatment arm to obtain sufficient effective sample size to detect an odds ratio of 1.66 at 12-month follow-up using a proportional odds regression model. The odds ratio gives the ratio of the odds that the patient portal intervention group has no diabetes care gaps over the odds that the usual care group has no gaps. Power estimates are based on an attrition rate of 20% at the time of study completion. The power analysis assumes that the distribution of diabetes care gaps is that 26% of the population had no gaps, 32% had 1 gap, 23% had 2 gaps, 14% had 3 gaps, and 5% had 4 gaps. This assumption was based on preliminary data obtained from VUMC during November 2021. The desired power is assumed to be 80% with a significance level of .05.

### Ethical Considerations

This study was approved by the Vanderbilt University institutional review board (IRB# 212257). Patient participation was voluntary, and patients provided informed consent prior to their participation. REDCap electronic data capture tools hosted at Vanderbilt University were used to collect and manage study data [37,38]. REDCap is a secure, HIPAA-compliant, web-based software platform developed to support data capture and management for research studies. REDCap access was limited to the minimum number of study team members needed to conduct the trial. Participants are compensated US $40 for completing each study questionnaire (US $160 total if they complete all 4).

## Results

**Figure 2:**
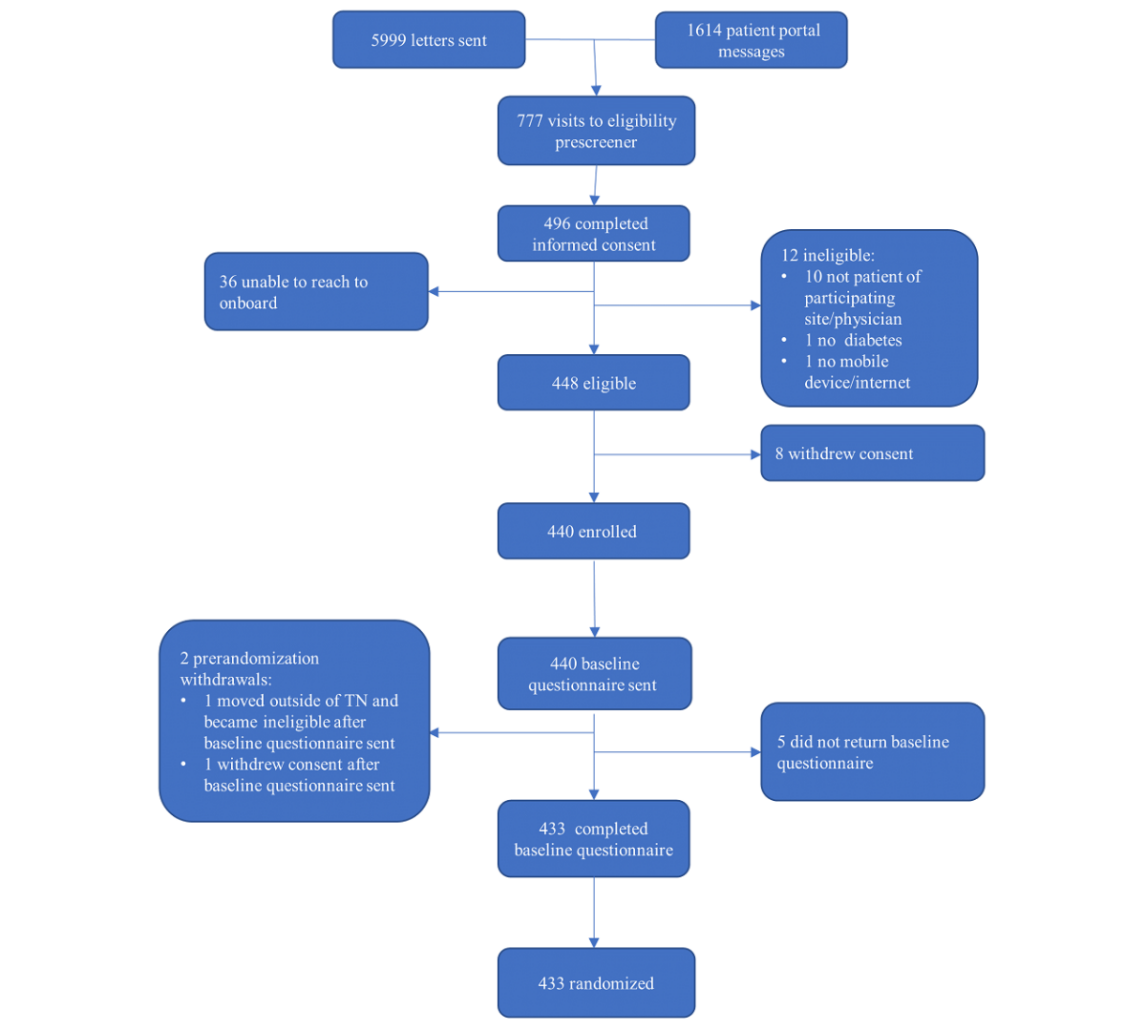
Recruitment and enrollment flow chart. TN: Tennessee.

Figure 2 shows a flow chart of recruitment. Recruitment began in April 2022 and ended in January 2023. Throughout the recruitment period, 5999 unique letters were sent to patients identified as potentially eligible. Separately, 1614 patient portal messages were sent to patients who use MHAV. Because it was not possible for the study team to cross-reference the list of those who were sent letters against the list of those who were sent patient portal messages, some overlap between the 2 is possible. The letters and emails generated 777 visits to the web-based REDCap eligibility prescreener and resulted in 496 completed informed consent documents. Among those who completed the informed consent document, 2.4% (n=12) were ineligible, 7.3% (n=36) were unreachable, and 90.3% (n=448) were confirmed eligible. Eight eligible patients declined to participate. All remaining eligible patients (n=440) were sent the baseline questionnaire. A total of 433 participants completed the baseline questionnaire and were randomized.

Of those randomized (n=433), the majority 66.5% (n=288) of participants were non-Hispanic White, and 33.5% (n=145) were from minoritized racial or ethnic groups. In addition, 33.9% (n=147) were aged 65 years or older. Furthermore, 8.8% (n=38) reported educational attainment of a high school degree or less, 30.7% (n=133) indicated limited health literacy, and 26.8% (116/433) had only a US governmental health plan (eg, military, Civilian Health and Medical Program of the Uniformed Services, Veterans Affairs, Medicaid, and Medicare).

## Discussion

### Principal Findings

This paper presents the protocol for a randomized trial evaluating the impact of a patient portal intervention empowering patients to address diabetes care gaps on the completion of selected evidence-based diabetes monitoring and preventive services. We aim to directly test the hypothesis that a patient portal intervention—alerting patients about selected diabetes care gaps, fostering understanding of their significance, and allowing patients to initiate orders for the corresponding care—will reduce diabetes care gaps compared with usual care. The study addresses an important need to find easily scalable and sustainable solutions to improve the rates of diabetes monitoring and preventive services that can reduce the incidence and severity of diabetes complications. By measuring attitudes toward managing diabetes and diabetes self-efficacy, we will also assess whether empowering patients to address their own care gaps has the secondary benefit of increasing their confidence to manage their disease. The study will also collect data on various demographic variables, including age, health literacy, education, and eHealth literacy. These variables will be analyzed to explore their potential predictive value in relation to intervention outcomes. Because the intervention, if successful, could be extended to address other important care gaps (eg, cancer screening), the study has broad implications for chronic disease management and disease prevention.

### Comparison to Prior Work

Patient portal functionality, which allows patients to self-schedule appointments and initiate clinical services, is an emerging concept in health care. A recent review focused on the barriers and facilitators of patient self-scheduling in health care [39]. While most studies in the review focused on self-scheduling general health services, the DCGI aims to enhance the understanding and completion of disease-specific health services. In a recent retrospective observational study, Hahn et al [19] evaluated a patient portal reminder for colorectal cancer screening with an embedded order button allowing patients to self-order fecal immunochemical test kits. Participants who used the order button received their kits promptly, while those who did not were sent kits by mail at a later date. The authors found that patients who used the order button were nearly 4 times more likely to complete the kit suggesting that self-ordering may act as a commitment device. Our study builds on this work and is the first RCT to our knowledge to evaluate the effect of a patient portal “self-order” system on diabetes care gaps.

### Limitations

The study has important limitations. Our study is limited to diabetes monitoring and preventive services data available in the VUMC EHR and may not be a complete record of all diabetes care received by patients. In addition, our study relies on self-reported measures of several secondary outcomes that are subject to recall and social desirability bias. However, these validated, widely accepted measures offer the advantage of being less costly and burdensome than more objective measures. Our study is powered to examine the effects of the intervention on the number of diabetes care gaps per patient. Analyses examining the effects of the DCGI on other secondary outcomes (eg, glycemic control) and comparing its effects among subgroups (eg, patients with limited health literacy) may be informative but underpowered. We hope that this study will provide a rigorous level of evidence for the effect of empowering patients to address diabetes care gaps via a patient portal. The current availability of the intervention is limited to English. This decision was made to ensure the feasibility of designing and successfully conducting the initial trial. However, it is important to note that Spanish-speaking groups are disproportionately affected by diabetes [3]. Thus, a crucial goal is to translate the DCGI into Spanish and other languages. While patient portal interventions offer a variety of advantages, they may be affected by inequities in patient portal adoption, potentially resulting in fewer patients from minoritized groups benefiting from them [40]. Encouragingly, recent data indicate that patient portal adoption is on the rise among diverse patient groups, and with appropriate design, patient portals may reduce health disparities [41,42]. A notable strength of our study is the diversity of our sample, which includes 33.5% (n=145) of patients from minoritized racial or ethnic groups.

### Conclusions

The study aims to investigate the impact of a patient portal intervention empowering patients to address diabetes care gaps. We seek to understand not only whether the intervention reduces the number of care gaps per patient but also whether it increases patients’ confidence to manage their diabetes. The study will provide important insights that can be applied to design and implementation of interventions to address a broad range of care gaps, including cancer screening and vaccinations. These research findings, along with evolving patient portal functionality, will inform effective, scalable, and sustainable ways to engage patients in chronic disease management and disease prevention.

## Appendix

Multimedia Appendix 1 Peer-review report by the National Institute of Diabetes and Digestive and Kidney Diseases Special Emphasis Panel.

## Abbreviations

DCGI: diabetes care gap intervention
DM: diabetes mellitus
EHR: electronic health record
MHAV: My Health at Vanderbilt
RCT: randomized controlled trial
REDCap: Research Electronic Data Capture
VUMC: Vanderbilt University Medical Center

## Data Availability

The data sets generated and analyzed during this study are available from the corresponding author on reasonable request.
