# Supplementary material for: Effects of a Patient Portal Intervention to Address Diabetes Care Gaps: Protocol for a Pragmatic Randomized Controlled Trial
Source: JMIR Res Protoc. 2024 Jun 28;13:e56123. doi: 10.2196/56123 (PMC11245660; doi:10.2196/56123)
Supplement: Multimedia Appendix 1 [file resprot_v13i1e56123_app1.pdf]

SUMMARY STATEMENT

PROGRAM CONTACT: (Privileged Communication) *Release Date:* 05/28/2019
Revised Date:

[redacted]

Application Number: 1 R18 DK123373-01

Principal Investigator

MARTINEZ, WILLIAM

Applicant Organization: VANDERBILT UNIVERSITY MEDICAL CENTER

Review Group: ZDK1 GRB-1 (O1)
National Institute of Diabetes and Digestive and Kidney Diseases Special Emphasis Panel
Pragmatic Research and Natural Experiments

Meeting Date: 05/17/2019
Council: OCT 2019
Requested Start: 12/01/2019

RFA/PA: PAR18-925
PCC: DHB BIT2

Project Title: Expanding Patient Engagement in Diabetes Care: Patient Portal Innovation

SRG Action: Impact Score:24
Next Steps: Visit https://grants.nih.gov/grants/next_steps.htm
Human Subjects: 30-Human subjects involved - Certified, no SRG concerns
Animal Subjects: 10-No live vertebrate animals involved for competing appl.
Gender: 1A-Both genders, scientifically acceptable
Minority: 1A-Minorities and non-minorities, scientifically acceptable
Age: 7A-Only Adults, scientifically acceptable

Project Year	Direct Costs Requested	Estimated Total Cost
1	[redacted]	[redacted]
2		
3		
4		
5		
TOTAL	[redacted]	[redacted]

ADMINISTRATIVE BUDGET NOTE: The budget shown is the requested budget and has not been adjusted to reflect any recommendations made by reviewers. If an award is planned, the costs will be calculated by Institute grants management staff based on the recommendations outlined below in the COMMITTEE BUDGET RECOMMENDATIONS section.

1R18DK123373-01 Martinez, William

BUDGETARY OVERLAP

RESUME AND SUMMARY OF DISCUSSION: This application was submitted by Dr. William Martinez on behalf of Vanderbilt University in response to Program Announcement PAR-18-925, "Pragmatic Research in Healthcare Settings to Improve Diabetes and Obesity Prevention and Care (R18)." The proposed study will evaluate strategies to support primary care physicians in evidence based monitoring to attain benchmarks of diabetes care. The intervention is focused on an EPIC based patient portal that will notify patients when diabetes monitoring and preventive services are due; there is also the capacity for patients to self-order a lab or service and self-schedule visits for preventive services. Strengths of the application include the potential for the proposed technology to improve self-management; the multidisciplinary team, which is also supported by the Vanderbilt CDTR; evidence of feasibility for the proposed intervention; and the potential for dissemination through EPIC. Although the remarkably high rate of engagement in the patient portal at Vanderbilt is a strength for this study, there is also a concern that this might impact generalizability. Minor concerns include an uncertainty whether the intervention would be provided to other systems as a free product; the potential for differential uptake in some high-risk populations; minor inconsistencies in how recruitment shortfalls will be addressed; and the use of a single site for the study. Overall, strengths outweighed weaknesses and the application was judged to be excellent to outstanding.

DESCRIPTION (provided by applicant): Evidence-based diabetes monitoring & preventative services can prevent or delay many costly and highly morbid disease-related complications, yet many patients do not receive all clinically-meaningful, evidence- based services. For example, while detecting and treating early diabetic eye disease can reduce the development of severe vision loss by an estimated 60%, approximately 40% of Americans with diabetes do not receive an annual eye exam. Prior research has demonstrated numerous barriers to the completion of evidence-based diabetes monitoring & preventative services including patient factors (e.g., lack of awareness and limited health literacy) and clinician/system factors (e.g., limited physician time and patient support between visits). Attempts to increase diabetes monitoring & preventative services have had only modest results. To achieve optimal rates of these services, an intervention is urgently needed that improves clinical efficiency by decreasing clinician workload, is appropriate for patients with varying levels of health literacy, and is highly scalable and sustainable. By providing an engaging and convenient means to track and visualize health data, obtain education and guidance, receive notifications, and connect patients and doctors, patient portals offer a promising platform to enhance access to health services while overcoming the limitations of costly and difficult to scale face-to-face interventions. Our research team previously applied user-centered Design Sprint methodology to develop patient portal features and functionality that were engaging and satisfying for a diverse group of patients with diabetes to use and provided users with a better understanding of their diabetes health data (e.g., A1C). Feedback from users indicated a desire to self-order and self-schedule services when they become due. Using a similar approach and with strong institutional endorsement, we will design, usability test, and evaluate a novel patient portal intervention to: (a) notify patients when selected, clinically-meaningful, evidence-based diabetes monitoring & preventative services (e.g., annual eye exam) become due and provide reminders for timely completion, (b) promote understanding of the importance of these services through literacy sensitive content, and (c) allow patients, when due by evidence-based guidelines, to ‘self-order’ (requires primary care physician (PCP) single-click co-signature) lab tests (e.g., A1C) and vaccines prior to doctor’s visits and self-schedule clinical services (e.g., eye exam). Specifically, we aim to: (1) apply Design Sprint methodology and usability testing to design and test a patient portal intervention (described above) that can overcome patient and clinician/system barriers to completing evidence-based, clinically meaningful, diabetes monitoring & preventative services among a diverse group of patients, (2) evaluate the effect of the patient portal intervention developed in Aim 1 on the completion of those services in a pragmatic, cluster randomized controlled trial, and (3) assess PCP’s attitudes and

experiences, through surveys and interviews, regarding the acceptability and usefulness of the intervention and its impact on clinical efficiency.

PUBLIC HEALTH RELEVANCE: Evidence-based diabetes monitoring & preventative services can prevent or delay many costly and highly morbid disease-related complications, yet many patients do not consistently receive all evidence-based, clinically meaningful services (e.g., annual eye exam). By providing a convenient and engaging means to track and visualize health data, obtain education and guidance, receive notifications, and connect patients with their healthcare team, patient portals can enhance access to health services while overcoming the limitations of costly and difficult to scale face-to-face interventions. The overall objective of this project is to design, usability test, and evaluate an innovative patient portal intervention (that includes functionality allowing patients, when due by evidenced-based guidelines, to self-order and self-schedule services) as a means of engaging patients in diabetes care and improving the completion of evidence-based diabetes monitoring & preventative services.

CRITIQUES: The written critiques of individual reviewers are provided in essentially unedited form below. These critiques were prepared prior to the meeting and may not have been revised afterwards. The "Resume and Summary of Discussion" above summarizes the final opinions of the committee.

CRITIQUE 1

Significance: 2
Investigator(s): 2
Innovation: 2
Approach: 1
Environment: 1

Overall Impact: Strategies are needed to support PCPs in obtaining all evidence-based diabetes monitoring and preventative services as a first step in facilitating 'moving the needle' in attainment of benchmarks for care, -, A1c must be obtained before clinical action steps can be taken to enable reaching benchmarks for care. The "Expanding Patient Engagement in Diabetes Care: Patient Portal Innovation" proposes to examine a patient portal intervention's impact on meeting all evidence-based diabetes monitoring and preventative services. The study team will design, usability test, and evaluate the intervention which will be built for the My Health at Vanderbilt (MHAV) EPIC patient portal which is in use among primary care practices in the Vanderbilt health system to: (a) notify patients when selected, evidence-based diabetes monitoring & preventative services (e.g., annual eye exam) are due and provide reminders for timely completion, (b) promote patient understanding of the importance of these services, and (c) allow patients, when due, to 'self-order' , with PCP single-click co-signature, lab tests (e.g., A1c) and vaccines prior to doctor's visits and self-schedule clinical services (e.g., eye exam). Will: (1) apply Design Sprint methodology and usability testing to design and test the portal intervention to support overcoming barriers to completing evidence-based, clinically meaningful, diabetes monitoring & preventative services among a diverse group of patients, (2) evaluate the effect of the patient portal intervention on the completion of preventive services in a pragmatic, cluster randomized controlled trial, and (3) assess PCP's attitudes and experiences regarding the acceptability and usefulness of the portal intervention and its impact on clinical efficiency. Technology offers promise for engaging patients in self-care related activities. As patient portals integrated into EHRs gain traction they can be used to facilitate engagement with the patient in these activities. Automated strategies such as the proposed self-orders and self-scheduled clinic services which will be designed and implemented in this application could potentially take us one step closer to recognizing the potential of patient portals in helping to support both the patient and providers in assuring that DM monitoring and recommended

preventative services are obtained. This multi-disciplinary team which is supported by the VUMC CDTR appears to have the expertise (clinical, IT, biostats, etc.) to conduct the proposed work.

1. Significance:

Strengths

- The proposed research, which uses the eHealth Enhanced CCM, will meaningfully inform healthcare practice addressing an important gap in our knowledge as to how technology (specifically a patient portal that is integrated with the EPIC EHR) can support engaging patients and PCPs in assuring that adults with type 1 or type 2 diabetes have recommended maintenance and preventative care services carried out.
- This work could be used to justify policy which allows patients to self-order lab tests and vaccines, and to self-schedule related clinical services (with a one-click provider authorization).
- While this functionality is being built for EPIC which currently has a large share of the US EHR market, it could presumably be spread to other health systems which use EPIC and the concept applied in other EHRs if the study is successful.
- The intervention will be fully automated, thus saving the health system and providers from allocating effort to identify patients needing the services, ordering the tests and scheduling related services.
- Also assessing patient reported outcomes (activation, self-efficacy, DM knowledge, DM distress) using validated surveys.

Weaknesses

- Not all health systems have as robust engagement with their patient portal as the MHAV; however, over time this is likely to change which would further improve generalizability of the findings.

2. Investigators:

Strengths

- Strong team with track record of HSR and publications, grants individually and together; complementary multi-disciplinary skills which will support all aspects of study conduct.
- Martinez, PI, Asst Prof Med, Vanderbilt Health Services Research group – expertise in patient-facing technologies to engage DM patients in care and improve outcomes; Design Sprint and human factors evaluation methods;
- Rosenbloom directs Vanderbilt patient portal and research focuses on how patients and healthcare providers interact with health information technologies.
- Elasy Dir VU CDTR. With expertise in PROs, QI for system redesign and predictors of maintenance care.
- Mayberry is Psychologist with experience conducting mixed-methods research on use of MHAV portal.
- Hackstadt Leads Biostats core in VU CDTR. Focus on applications of novel statistical methods to different areas including HSR and diabetes
- Threatt UX Designer in Health IT Dept. Interest in how tech affects patient care – iterative design and evaluation processes.

Weaknesses

- None identified.

3. Innovation:

Strengths

- This will be first study to look at using self-ordering and self-scheduling via a patient portal for persons with diabetes. Will link orders to most recent DM diagnosis code used in chart for

purposes of billing and reimbursement. Will potentially serve to engage DM patients in self-care management.

- Use of **automated outreach to patients** with diabetes with reminders to have maintenance and preventative services ordered, helping to lighten the workload of PCPs and their staff in assuring benchmarks for care metrics are met so that appropriate care measures can be implemented as needed based on results.
- Detailed plan for design (Design Sprint) and for heuristic evaluation in first phases of study to assure usability
- Final phase longitudinal evaluation of impact of the intervention once designed, built and pilot tested (n=250) will evaluate services completion, PROs and A1c and DM and BP meds treatment intensification – all of which will be extracted directly from the EHR.
- If successful, the intervention can be spread for sustained use throughout a system or systems which use EPIC.
- As NIH funding would be supporting design, build and testing of this MyChart application it would be <u>of interest to obtain a commitment from EPIC</u> to allow use of the products by all MyChart users without accrual of significant additional costs to the user systems. If the apps/functionalities which are built under auspices of NIH funding for this study are to be made available in the EPIC app Orchard, will they be free or will there be a charge for other EPIC systems to use?
- Will use some features designed to engage users such as elements of game design and competition – which receive a fair bit of attention but for which evidence of impact is needed and will potentially be generated by this study.

Weaknesses

- Reference to some functionality in place in EPIC which can already accommodate self-ordering and scheduling by patients – as has been shown to be useful in increasing colonoscopy screenings. Will this existing infrastructure be used by the study to prevent duplication of effort/expense.
- Dependent upon uptake and engagement of the patient portal by patients with DM – do address populations which may be less likely to be tech adopters in effort to understand how will do with low literacy and older patients, etc.

4. Approach:

Strengths

- This study will support 3 aims: design and usability testing; pragmatic, parallel-design, cluster RCT; and assessment of PCP attitudes and experiences with the intervention – each of which is well described in detail in the application.
- The MHAV has very high levels of patient enrollment in their patient portal which should facilitate patient recruitment.

Weaknesses

- Pragmatic Cluster RCT with PCP as randomization target rather than clinics. Investigators acknowledge that this may be a weakness, but provide justification based on biostats considerations for why this was necessary.
- One item validated health literacy scale which queries confidence in filling our medical forms – apparently correlates with S-TOFHLA.
- Recruitment from a single academic medical center and some minor inconsistencies throughout the application in regard to how recruitment shortfalls will be addressed, e.g., referral of patients from larger CDTR studies or from patients who expressed interest in research (both of which could potentially confound generalizability in the long run and/or lead to underrepresentation of key demographics who might not be easily engaged in research but certainly need the service) versus approaching PCPs to help with recruitment/identification.

- Cost analysis limited to technology costs.
- No detail around gamification/competition features of approach provided
- Sub-analyses for the low literacy and older participants would be of interest/important to include in analyses.

5. Environment:

Strengths

- VUMC with robust environment including CDTR and Health IT and HSR groups who will be engaged in the study.
- Portal engagement high among patients in system.

Weaknesses

- Study to be conducted in one center which may impact generalizability. Would suggest considering adding additional site(s) from system, e.g., is stated there are urban and suburban clinics – would be useful to include at least one clinic from each setting, if not two.

Study Timeline:

Strengths

- **Study timeline table and description** provided seems feasible

Weaknesses

- None noted.

Sustainability:

Strengths

- Intervention is integrated into medical practices via embedded into the EMR patient portal used by the institution.
- Will utilize services of two existing FT IT staff who support VUMC MyChart portal
- Sr VP Quality and Safety LOS states will sustain beyond the grant period at VUMC
- Scalability, sustainability and dissemination potential among all users of EPIC nationally is discussed
- Reimbursement for services ordered is addressed in plan to auto link most recent DM dx

Weaknesses

- Implementation process for rolling out the intervention to staff and to patients is not well described. This will be important in informing efforts to spread beyond the study setting
- Cost associated with providing the intervention functions to EPIC users in other institutions is not discussed

Milestone Plan:

Strengths

- Measures align with the eHealth enhanced CCM model
- Primary and secondary outcomes clearly delineated in Table 2 and are highly appropriate for the study. Includes services completion; patient activation; PCP-patient communication; PROs; satisfaction with the portal; lab, vaccine and eye exam orders; eye exams scheduled; A1c and meds intensification.
- It will be of secondary interest to the diabetes community to see if the clinical outcomes (BG control/A1c and treatment intensification for DM and HTN) are improved if the proportion of the five selected DM and monitoring and preventative services (annual A1c, nephropathy screening, flu vaccine, DM eye exam and pneumococcal vaccine) is increased by the intervention.

Weaknesses

- It would also be important to know that the tests and vaccines ordered have been done/administered/incorporated into the patient chart and signed off on by the PCP to show that they were completed appropriately.
- And that patients have been informed of the results (via the portal)

Protections for Human Subjects:

Acceptable Risks and/or Adequate Protections

- Low risk technology intervention

Data and Safety Monitoring Plan (Applicable for Clinical Trials Only):

Acceptable

Full plan provided.

Inclusion of Women, Minorities and Children:

- Sex/Gender: Distribution justified scientifically
- Race/Ethnicity: Distribution justified scientifically
- For NIH-Defined Phase III trials, Plans for valid design and analysis: Not applicable
- Inclusion/Exclusion of Children under 18: Excluding ages <18; justified scientifically
- To account for the unique usability challenges of older patients and those with limited health literacy, we will purposively sample participants to ensure study samples contain a minimum of 20% of patients with limited health literacy and 20% over 65.

Vertebrate Animals:

Not Applicable (No Vertebrate Animals)

Biohazards:

Not Applicable (No Biohazards)

Budget and Period of Support:

Recommend as Requested

Recommended budget modifications or possible overlap identified:

- 4 'other' personnel for 32.4 cal months totaling [redacted] = Project Coordinator, Research Asst; Sr Programmer & masters level biostatistician. (All okay)
- MTDC DHHS [redacted] (p47/180) - what does this represent? in budget justification

.**Resource Sharing Plans:**

Acceptable

- Plan provided for access to data generated by the study

Additional Comments to Applicant (Optional):

- Strong application with only a few minor modifications needed.

CRITIQUE 2

Significance: 2
Investigator(s): 2
Innovation: 3
Approach: 3

Environment: 1

Overall Impact: This R18 application outlines the design, usability testing and evaluation of a patient portal intervention for patients with diabetes to improve adherence to guideline-concordant diabetes measures (primary outcome). The first study aim is to design and suability test the patient portal intervention, building upon the PI's prior K23-funded work in developing a patient-facing diabetes dashboard. Next, the patient portal intervention will be evaluated in a pragmatic, cluster randomized controlled trial to determine completion of evidence-based diabetes monitoring and preventive services (primary outcome) and secondary intermediate outcomes. Lastly, PCP attitudes and experiences about the acceptability and usefulness of the intervention as well as impact on clinical efficiency will be assessed using qualitative methods. The proposed study is significant and innovative, building logically upon the PI's prior work to engage patients with diabetes to improve their guideline-concordant care. Further, the patient outcomes selected are clinically meaningful, and if effective, the intervention should easily garner support from healthcare system leadership interested in pursuing value-based care. The PI has assembled a multidisciplinary team with the appropriate expertise and experience to conduct the proposed work. A minor weakness is concern about the ability to recruit the necessary patients (500) within the proposed timeframe. The research environment is outstanding and letters of support suggest leadership engagement and opportunity for sustainability if the intervention is found to be effective.

1. Significance:

Strengths

- Only approximately 50% of patients nationally receive guideline-concordant diabetes monitoring and preventive services. Adherence to these services can prevent long-term complications, indicating a need to identify approaches to improve these rates.
- Patient portals represent an existing platform from which to develop interventions to increase patient engagement in their diabetes care. Technological advances now allow for self-scheduling and self-ordering, which may improve adherence to services if utilized as proposed.
- Understanding approaches to improve diabetes monitoring and preventive service uptake is of great value to institutions as reimbursement approaches shift to value-based care. This allows for institutional support for successfully-integrated interventions. Utilization of the existing patient portal is a major strength in allowing study results to meaningfully inform healthcare practice.
- The vast majority of patients are interested in utilizing web-based interventions for healthcare, suggesting patient portals may be of increasing interest. Further, the underlying EHR system engages half of Americans, providing significant opportunity for dissemination to other healthcare settings if successful.

Weaknesses

- Web-based interventions may be less utilized by older adult populations, who make up a significant percent of patients with diabetes.

2. Investigators:

Strengths

- Dr. Martinez is a clinician-investigator and primary care physician with a research focus on innovating to improve healthcare quality and safety, specifically utilizing health apps. His K23 award forms the basis of the proposed study intervention, which successfully utilized Design Sprint methodology to develop a patient-facing diabetes dashboard embedded in the patient portal, providing him with the expertise and experience to oversee the proposed study.
- Dr. Martinez has assembled as multidisciplinary team with the expertise to conduct the proposed work. Further, he has previously collaborated and successfully published with this team. Team members include Drs. Rosenbloom (directs Vanderbilt's patient portal; expertise in biomedical informatics), Elasy (Division Director, Director of Vanderbilt's Center for Diabetes

and Translation Research, expertise in diabetes research), Mayberry (expertise in mixed-methods research, Director of Effective Health Communication Core), Hackstadt (Directs Vanderbilt's Center for Diabetes and Translational Research Biostatistical Core) and Threatt (Senior UX Designer at Vanderbilt's HealthIT Department).

Weaknesses

- None noted.

3. Innovation:

Strengths

- Use of the patient portal, with integration of new technologies of allowing patient's to self-order and self-schedule, is an innovative approach to improve value-based care for patients with diabetes.
- Utilization of the Design Sprint approach, successfully deployed in the PI's preliminary work, will help to ensure usability and acceptability of the proposed intervention.
- Integration into the existing patient portal, which utilizes the mostly widely-adopted EHR platform, will offer opportunity for dissemination if found to be successful.

Weaknesses

- None noted.

4. Approach:

Strengths

- Aim 1: The investigative team clearly outlines how they will utilize user-centered Design Sprint methodology to achieve Aim 1's goal of designing an acceptable intervention. Participant eligibility criteria has been minimized to ensure a generalizable sample. Data collection and measures utilize validated measures and procedures by investigators with appropriate expertise and experience. Two studies will be completed under this aim, including a longitudinal prospective usability study to ensure longer-term usability and engagement. Qualitative studies are supported by the Qualitative Research Core.
- Aim 2: Study design to evaluate the effectiveness of the patient portal intervention on the completion of selected evidence-based, clinically-meaningful, diabetes monitoring and preventive services will be a pragmatic, parallel-design, cluster randomized controlled trial. Cluster design is appropriate to minimize contamination as a result of PCPs changing care due to engagement in the intervention arm. A pragmatic approach allows greater generalizability across patients with diabetes. Data outcomes and instruments as well as timeline for collection are validated and appropriate for the proposed measures.
- Aim 3: Assessment of PCP attitudes and experience as well as impact on clinical efficiency will be through a cross-sectional, mixed-methods approach. This is a strength of the proposed study, given PCP engagement and support will be important to the future success of this type of patient intervention.

Weaknesses

- A minor concern is that the proposed approach requires successful enrollment is greater than 5% of eligible patients in a recruitment period of approximately 9 months. This feels ambitious to recruit 500 patients. It is noted the research team has successfully recruited for similar prior studies, although over unclear timeframes.

5. Environment:

Strengths

- Vanderbilt has significant support for the proposed study. Specifically, the Vanderbilt Patient Portal is one of the best adopted portals in the country and under the leadership of Dr. Rosenbloom (Co-I).

- Subject recruitment will be assisted by Vanderbilt's Subject Locator database and MyResearch repository of patients. Further, Dr. Elasy oversees the 14 clinics which will be engaged in subject recruitment, which have identified over 8,000 potentially eligible patients.
- A letter of support is provided from leadership from Vanderbilt's CTSA (VICTR), offering several resources to the proposed study. Specifically, the qualitative study (aim 3) will be supported by the Vanderbilt Qualitative Research Core (letter of support provided).

Weaknesses

- None noted.

Study Timeline:

Strengths

- The study timeline is described in detail and takes into account appropriate start-up activities, rate of enrollment and planned follow-up assessments.
- The proposal incorporates multiple efficiencies from existing resources, including Vanderbilt's CTSA and existing work with the EHR/patient portal (i.e., PI's prior work).

Weaknesses

- As above, potential recruitment challenges noted. The recruitment plan does articulate engaging PCPs to increase recruitment, however, prior success with this approach is not described.

Sustainability:

Strengths

- A significant strength of the application is the sustainability plan, which describes excellent institutional support from key stakeholders. Specifically, the patient portal intervention would not require additional personnel to be maintained.
- Utilization of the most prevalent EHR platform is an additional strength of the application which offers tremendous opportunity for scalability and dissemination.
- If successful, the study's primary outcome is critical for value-based care and therefore important to healthcare system leadership. This outcome selection supports opportunity for sustainability and further dissemination if the intervention is successful.

Weaknesses

- Less than half of patients currently utilize the patient portal, which is the most adopted in the country. However, this remains a significant percentage of patients which could potentially benefit if found to be successful.

Milestone Plan:

Strengths

- Milestones are appropriate, meaningful, and feasible for the proposed work.

Weaknesses

- None noted.

Protections for Human Subjects:

Acceptable Risks and/or Adequate Protections

- The human subject protections plan was reviewed and found to be acceptable for this minimal risk study.

Data and Safety Monitoring Plan (Applicable for Clinical Trials Only):

Acceptable

The data and safety monitoring plan was reviewed and found to be acceptable for this minimal risk study.

Inclusion of Women, Minorities and Children:

- Sex/Gender: Distribution justified scientifically
- Race/Ethnicity: Distribution justified scientifically
- For NIH-Defined Phase III trials, Plans for valid design and analysis: Scientifically acceptable
- Inclusion/Exclusion of Children under 18: Excluding ages <18; not justified scientifically
    - Inclusion criteria reviewed. Appropriate to exclude children under 18 given the intervention and relevant outcomes would be different for this population.

Vertebrate Animals:

Not Applicable (No Vertebrate Animals)

Biohazards:

Not Applicable (No Biohazards)

Budget and Period of Support:

Recommend as Requested

Resource Sharing Plans:

Acceptable

CRITIQUE 3

Significance: 3
Investigator(s): 2
Innovation: 3
Approach: 2
Environment: 3

Overall Impact: The project is reasonably likely to exert a strong influence on the field of health informatics. Not clear that these particular intervention technologies will be of high interest to low-income and/or medically underserved patients of greatest need -additional pilot data would be of interest. There is some concern that the intervention may not be as valuable for low-income/low health literacy patients. Self-ordering feature has potential to be of great interest to patients and could be very empowering to patients to better understand and take charge of their care. Potential for investigators to integrate this intervention into a widely used EHR supplied by Epic Systems Corp. could allow dissemination of the intervention across multiple health systems and improve care for millions of patients.

1. Significance*:*

Strengths

- Very good potential to inform practice and policy.
- Potential for investigators to integrate this intervention into a widely used EHR supplied by Epic Systems Corp. (Verona, WI), with over 127 million patient records, could allow dissemination of the intervention across multiple health systems and improve care for millions of patients.

Weaknesses

- Not many health systems have as developed of a patient portal and it may be hard for other sites to replicate this work.
- Not clear that this intervention will be as likely to benefit low-income/low health literacy patients and not as clear that this intervention will improve PAM scores.
- No prespecified analysis of heterogeneity of treatment effects for vulnerable populations, for example, according to socioeconomic status, race/ethnicity, education level, health literacy, and/or technology use.

2. Investigators:

Strengths

- Very strong investigative team

Weaknesses

- PI is fairly junior.

3. Innovation:

Strengths

- Four component patient portal intervention highly innovative.
- Taking advantage of recent advances in patient portal technology to “engage patients further in their care by providing the infrastructure necessary to enable patients to self-schedule and ‘self-order’ health services” extremely innovative.
- Fairly innovative to use patient portal for reminders.

Weaknesses

- Complex intervention and impact of individual components of intervention difficult to assess given current methodology

4. Approach:

Strengths

- Approach for Aim 1 is excellent -very solid
- Aim 2 approach includes a usability study
- Cluster randomization at the physician level appropriate and should provide a very solid usual care control group.

Weaknesses

- Aim 3 not so compelling. Assessment of provider attitudes as part of third aim, while important, may not be as exciting or important as patient use and attitudes regarding different portal features.
- Presentation of two studies related to Aim 1 is confusing. Clearer differentiation of the “Task-based Usability Study of the Prototype” and the “Prospective, Longitudinal Usability Study of the Developed Product” would be helpful. However, this approach is very reasonable.

5. Environment

Strengths

- Very strong environment for this work. Solid data on baseline completion rates of evidence-based diabetes monitoring & preventative services among established primary care patients in the Vanderbilt system.
- Clear commitment of ongoing in-kind support from VUMC’s HealthIT Department for patient portal development activities

Weaknesses

- None

Study Timeline:

Strengths

- Study timeline described in detail, taking into account start-up activities, the anticipated rate of enrollment, and planned follow-up assessment.

Weaknesses

- None identified.

Sustainability:

Strengths

- Potential for investigators to integrate this intervention into a widely used EHR supplied by Epic Systems Corp. (Verona, WI), with over 127 million patient records, could allow dissemination of the intervention across multiple health systems and improve care for millions of patients. Investigators propose to use Epic's App Orchard to share the application programming interfaces (APIs) and create supporting materials but they do not indicate whether charges will apply.

Weaknesses

- Epic can be a difficult collaborator. Not clear if Epic or Vanderbilt are fully committed to integrating these tools into Epic and making them broadly available. Newly developed tools will be part of the My Health At Vanderbilt system and full assurances to make them available to other Epic users at low or no costs are not clearly made.

Milestone Plan:

Strengths

- Strong milestones

Weaknesses

- None identified

Protections for Human Subjects:

Acceptable Risks and/or Adequate Protections

- risks acceptable

Data and Safety Monitoring Plan (Applicable for Clinical Trials Only):

Acceptable

Data safety and monitoring committee for Studies 2 and 3.

Inclusion of Women, Minorities and Children:

- Sex/Gender: Distribution justified scientifically
- Race/Ethnicity: Distribution justified scientifically
- For NIH-Defined Phase III trials, Plans for valid design and analysis:
- Inclusion/Exclusion of Children under 18: Excluding ages <18; justified scientifically
    - Appropriate

Vertebrate Animals:

Not Applicable (No Vertebrate Animals)

Biohazards:

Not Applicable (No Biohazards)

Budget and Period of Support:

Recommend as Requested

Recommended budget modifications or possible overlap identified:

- No major overlaps identified. But given lower level of interest in third aim, NIH could consider a budget reduction to not fund work related to third aim.

Resource Sharing Plans:

Acceptable

CRITIQUE 4

Significance: 3
Investigator(s): 1
Innovation: 3
Approach: 3
Environment: 1

Overall Impact: This well-written application by Dr. Martinez and colleagues at Vanderbilt University proposes to develop and establish an enhanced patient portal that permits patients with diabetes to become more engaged in their care, and ultimately, close care gaps in uptake of preventive services to detect and prevent diabetes complications. The investigators propose to incorporate user perspectives in their design and testing phases, and then to test the effect of the portal system in a cluster-randomized trial of 94 primary care physicians, as well as gather qualitative data regarding user-friendliness, acceptability, and satisfaction among physicians. This component is aimed at evaluating whether the intervention reduces burnout and improve physicians' efficiency and workflow, which are currently important contemporary challenges in health services research. The ideas proposed are innovative and have the potential to substantially improve current clinical practice and comprehensiveness of meeting diabetes care goals. The merits notwithstanding, there were a few queries that the investigators ought to consider addressing: 1) providing clarity on where the aims of the PI's recently funded R03 end and where the aims of this R18 start; 2) considering the potential negative externalities of the intervention and how they will guard against these (for example, is there a chance that this tool increases physician burnout by increasing the need to be responsive to unnecessary testing requests?); 3) discussing whether and how the tool would be de-implemented if the intervention is not successful; and 4) discussing the potential pitfalls of the cluster RCT design and also providing more clarity on whether clinics or physicians are being randomized, and if the latter, is there a chance that there will be within-clinic contamination across physicians? I view this application as having potentially high scientific and also high real-life impact potential.

1. Significance:

Strengths

- Recognition that gaps in preventive and care services are multi-factorial
- The investigators also lay a clear rationale for why the user-centered design and testing are important
- The investigators' recognition of how this fits into routine healthcare delivery and the challenges and potential for impact are well justified

Weaknesses

- Of particular interest, the investigators argue that the shift in empowering patients to self-order and self-schedule improves the patients' engagement and also decreases physician burden – the data to support these assertions are a little limited, but the notion is compelling

2. Investigators:

Strengths

- Terrific multidisciplinary team including physicians, health services researchers, bioinformatics, biostatistics, psychology, and health IT
- In addition to expertise, each team member also brings unique reach into the health system, vendors, and also patients to be able to conduct this pragmatic research
- PI Martinez is an early stage investigator and clearly brings a unique set of perspectives and talents to answering this innovative set of questions

Weaknesses

- Impressed by the PI's productivity, but concerned that his newly funded R03 may have overlapping aims

3. Innovation:

Strengths

- Innovative idea and methods
- Important that the investigators have identified the context of implementation (e.g., physician burnout, etc.) and that they are cognizant of this in their design

Weaknesses

- There is not much discussion or thought around downside risk: What about potential over testing? Although the clinician has to co-sign, if the patients are demanding inappropriate testing, how does one negotiate over the portal? And does that then lower patient satisfaction and clinician satisfaction.

4. Approach:

Strengths

- The user-centered design phase in Aim 1 is innovative and appropriate – I would consider adding a few practicing physicians also to this phase as examining usability from patients alone seems to overlook the importance of the two-way communication and acceptability for this tool to work in practice
- The physician-user is the focus of Aim 3, but here again, I feel that examining the patient experience and perspective may also be important

Weaknesses

- Is compensation for participants in pragmatic research appropriate?
- Is a cluster RCT necessary? It seems that there would be no contamination if individuals are logging onto their accounts, unless those users are related or neighbors, etc.
- Why an R18 and not a standard R01? Seems like this is not translation of an already-proven intervention into health care settings. I can see the justification that the research design is pragmatic in that this technology is being developed and then tested within the healthcare system in pragmatic style
- Will the unit of randomization be the physician or clinic or both? If physicians, then will there not be intra-clinic contamination between physicians?
- 12-month follow up doesn't permit any flexibility for patients that sometimes get their preventive service in 15 months… would you consider?

5. Environment:

Strengths

- Strong environment, equipment, facilities, and mentors.

Weaknesses

- None noted

Study Timeline:

Strengths

- Timeline is described and appears reasonable

Weaknesses

- None

Sustainability:

Strengths

- Case for sustainability is well made and there are appropriate letters of support from health leaders and those that oversee the health system's patient portal

Weaknesses

- Unclear what the team will do to de-implement if the intervention has no additional value

Milestone Plan:

Strengths

- Milestones are described and appear reasonable

Weaknesses

- None

Protections for Human Subjects:

Acceptable Risks and/or Adequate Protections

Data and Safety Monitoring Plan (Applicable for Clinical Trials Only):

Acceptable

Inclusion of Women, Minorities and Children:

- Sex/Gender: Distribution justified scientifically
- Race/Ethnicity: Distribution justified scientifically
- For NIH-Defined Phase III trials, Plans for valid design and analysis: Not applicable
- Inclusion/Exclusion of Children under 18: Excluding ages <18; justified scientifically

Vertebrate Animals:

Not Applicable (No Vertebrate Animals)

Biohazards:

Not Applicable (No Biohazards)

Budget and Period of Support:

Recommend as Requested

Recommended budget modifications or possible overlap identified:

Resource Sharing Plans:

Acceptable

THE FOLLOWING SECTIONS WERE PREPARED BY THE SCIENTIFIC REVIEW OFFICER TO SUMMARIZE THE OUTCOME OF DISCUSSIONS OF THE REVIEW COMMITTEE, OR REVIEWERS' WRITTEN CRITIQUES, ON THE FOLLOWING ISSUES:

PROTECTION OF HUMAN SUBJECTS: ACCEPTABLE
INCLUSION OF WOMEN PLAN: ACCEPTABLE
INCLUSION OF MINORITIES PLAN: ACCEPTABLE
INCLUSION OF CHILDREN PLAN: ACCEPTABLE

BUDGETARY OVERLAP: There is potential scientific and budgetary overlap between this application and R03 DK119735, "Design Sprint and Usability Testing of a Patient-facing Diabetes Dashboard Embedded in an Existing Patient Portal Mobile App." In the event of an award, NIDDK staff must ensure that any potential overlap is resolved.

COMMITTEE BUDGET RECOMMENDATIONS: The budget was recommended as requested.

Footnotes for 1 R18 DK123373-01; PI Name: Martinez, William

NIH has modified its policy regarding the receipt of resubmissions (amended applications). See Guide Notice NOT-OD-14-074 at http://grants.nih.gov/grants/guide/notice-files/NOT-OD-14-074.html. The impact/priority score is calculated after discussion of an application by averaging the overall scores (1-9) given by all voting reviewers on the committee and multiplying by 10. The criterion scores are submitted prior to the meeting by the individual reviewers assigned to an application, and are not discussed specifically at the review meeting or calculated into the overall impact score. Some applications also receive a percentile ranking. For details on the review process, see http://grants.nih.gov/grants/peer_review_process.htm#scoring.
